# IBTK Haploinsufficiency Affects the Tumor Microenvironment of Myc-Driven Lymphoma in E-myc Mice

**DOI:** 10.3390/ijms21030885

**Published:** 2020-01-30

**Authors:** Eleonora Vecchio, Giuseppe Fiume, Chiara Mignogna, Enrico Iaccino, Selena Mimmi, Domenico Maisano, Francesco Trapasso, Ileana Quinto

**Affiliations:** 1 Department of Experimental and Clinical Medicine, University Magna Graecia of Catanzaro, 88100 Catanzaro, Italy; 2 Interdepartmental Service Center, University “Magna Graecia” of Catanzaro Medical School, 88100 Catanzaro, Italy

**Keywords:** tumor microenvironment, lymphoma, Myc, haploinsufficiency, heterozygous mutation, IBTK

## Abstract

The tumor microenvironment is a dynamic and interactive supporting network of various components, including blood vessels, cytokines, chemokines, and immune cells, which sustain the tumor cell’s survival and growth. Murine models of lymphoma are useful to study tumor biology, the microenvironment, and mechanisms of response to therapy. Lymphomas are heterogeneous hematologic malignancies, and the complex microenvironment from which they arise and their multifaceted genetic basis represents a challenge for the generation and use of an appropriate murine model. So, it is important to choose the correct methodology. Recently, we supported the first evidence on the pro-oncogenic action of IBTK in Myc-driven B cell lymphomagenesis in mice, inhibiting apoptosis in the pre-cancerous stage. We used the transgenic *Eμ-myc* mouse model of non-Hodgkin’s lymphoma and *Ibtk* hemizygous mice to evaluate the tumor development of Myc-driven lymphoma. Here, we report that the allelic loss of *Ibtk* alters the immunophenotype of Myc-driven B cell lymphomas, increasing the rate of pre-B cells and affecting the tumor microenvironment in *Eμ-myc* mice. In particular, we observed enhanced tumor angiogenesis, increasing pro-angiogenic and lymphangiogenic factors, such as VEGF, MMP-9, CCL2, and VEGFD, and a significant recruitment of tumor-associated macrophages in lymphomas of *Ibtk^+/-^* *Eμ-myc* compared to *Ibtk^+/+^* *Eμ-myc* mice. In summary, these results indicate that *IBTK* haploinsufficiency promotes Myc tumor development by modifying the tumor microenvironment.

## 1. Introduction

The chemokine system plays a key role in cancer-related inflammation involved in all stages of tumor development. Noteworthy, chronic inflammation in the tumor microenvironment contributes to the progression of many cancer types [1,2,3]. It is widely recognized that chronically activated immune cells sustain tumor growth and survival. However, the underlying mechanisms of this connection remain unclear. The tumor microenvironment consists of different cell populations, including fibroblasts, endothelial cells, and immune cells [4,5]. It is now clear that chronically activated immune cells can promote tumor growth and facilitate tumor survival. Macrophages are typically the main inflammatory component, but a variety of tumor-infiltrated immune cells can participate in tumor promotion [6]. In general, the presence of macrophages in tumor tissues confers a poor prognosis [7]. Furthermore, angiogenesis is also a critical process in malignant progression [8]. Tumor growth is strictly associated with the expansion of vasculature that supplies oxygen and nutrients to the tumor tissue and allows metastatic spread to colonize distant organs. It was shown that the tumor microenvironment (TME) composition as well as its functionality are tissue dependent and context dependent [9]. A useful approach to understand the impact of TME on tumor evolution is the genetically engineered mouse model (GEMM). GEMMs are a powerful tool to reveal multiple genes implicated in the genesis of lymphoma and are extensively used to validate oncogene or tumor suppressor function [10,11]. Whereas each mouse model may clarify the molecular mechanism of lymphoma, evaluation schema for mouse tumors have not yet been standardized [10].

Myc is a transcription factor regulating the expression of genes involved in cell growth and metabolism, and it is one of the most frequently dysregulated transcription factors in human malignancies [12]. Over expression of Myc is a well-characterized initiating step in human Burkitt’s lymphoma, a non-Hodgkin’s lymphoma [13]. The *Eμ-myc* transgenic mouse is a pre-clinical model of human non-Hodgkin’s lymphoma, which develops aggressive B cell-derived lymphomas at an early age, with a 90% mortality rate by 20 weeks of age and median age of death at 12 weeks [13,14]. Myc^Tg^ lymphomas develop from the B220^low^ pre-B and B cell populations, and *Ig* gene rearrangement analyses indicate that most are monoclonal [13,15]. Lymphomas developed from *Eμ-myc* transgenic mice present increased blood and lymphatic vascular growth in secondary lymphoid organs [16]. 

The human *IBTK* gene is involved in the stress response and tumor growth [17,18]. It expresses a main protein IBtkα isoform, encoding a substrate receptor of Cullin3 ligase that promotes the proteasome-associated degradation of tumor repressor PDCD4 [19]. *IBTK* RNA interference affected the wide genome expression and RNA splicing in a cell-type-specific manner [20]. *IBTK* was hyper-expressed in chronic lymphocytic leukemia correlating with disease progression, and it was required for B cell survival upon stress induced by chemotherapeutic agents [21]. Based on the strong homology between the human and murine *IBTK* gene, we previously developed *Ibtk* knockout mice to address the role of *IBTK* in B-lymphomagenesis [22]. By taking advantage of the *Eμ-myc* transgenic mouse, we generated *Ibtk^-/-^Eμ-myc* offspring to support the first evidence of the pro-survival action of *IBTK* in Myc-dependent B-lymphomagenesis counteracting apoptosis of pre-cancerous B-cells [22]. In the present study, the *IBTK* haploinsufficiency alters tumor development and, consequently, the tumor microenvironment by enhancing tumor vascularization in Myc-driven B cell lymphoma. Allelic loss of *IBTK* promotes the expression of pro-angiogenic and inflammatory cytokines as VEGF family proteins together with the recruitment of tumor-associated macrophages (TAMs) as immune cells in Myc-driven lymphoma. These results contribute to the characterization of *IBTK* as a novel regulator gene of the tumor microenvironment.

## 2. Results

### 2.1. IBTK Haploinsufficiency Increases the Size and Vascularization of Spleen and Lymph Nodes of Eμ-myc Tumor Mice

*Eμ-myc* transgenic mice are widely used as a preclinical model of Myc-dependent B-lymphomagenesis [13,14]. We previously investigated the contribution of *IBTK* to malignant transformation of B cells by crossing *Eμ-myc* mice with *Ibtk^-/-^* mice to generate *Ibtk^-/-^ Eμ-myc* offspring [22]. While the complete loss of *Ibtk* (*Ibtk^-/-^Eμ-myc* mice) delayed the lymphoma onset and increased the lifespan, the loss of a single allele of *Ibtk* (*Ibtk^+/-^ Eμ-myc* mice) did not significantly affect tumor onset and the median age of mortality in *Eμ-myc* mice littermates [22].

**Figure 1:**
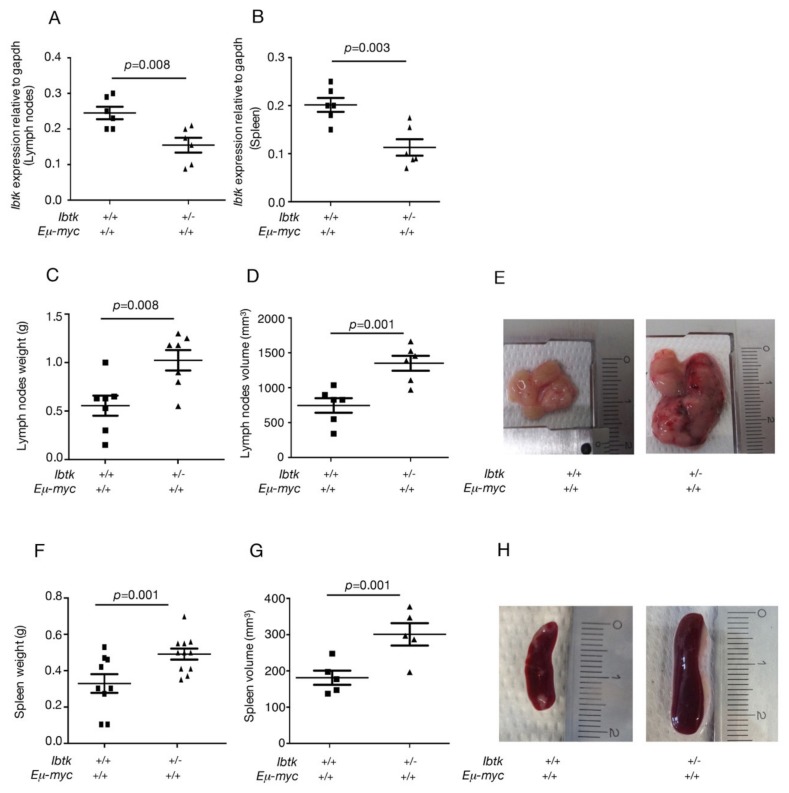
*IBTK* haploinsufficiency promotes the enlargement of tumor lymph nodes and spleen in *Eμ-myc mice*. **A,B.** *Ibtk* mRNA levels were measured by RT-PCR in the tumor lymph nodes and spleen of *Ibtk^+/+^ Eμ-myc* and *Ibtk^+/-^ Eμ-myc* mice, and normalized to *Gapdh* mRNA. **C**. Weights of lymph nodes of *Ibtk^+/+^Eμ-myc* and *Ibtk^+/-^ Eμ-myc* sick mice. Values are the mean ± SEM (*n* = 7/genotype). **D.** Volume of lymph nodes of *Ibtk^+/+^ Eμ-myc* and *Ibtk^+/-^ Eμ-myc* sick mice. Values are the mean ± SEM (*n* = 6/genotype). **E.** Representative morphology of tumor lymph nodes. Scale bar is indicated. **F.** Weights of spleens of sick lymphoma-burdened *Ibtk^+/+^ Eμ-myc* and *Ibtk^+/-^ Eμ-myc* mice. Values are the mean ± SEM (*n* = 10/genotype). **G.** Volume of spleens of sick lymphoma-burdened *Ibtk^+/+^ Eμ-myc* and *Ibtk^+ -^Eμ-myc* mice. Values are the mean ± SEM (*n* = 5/genotype) **H.** Representative morphology of tumor spleens. Scale bar is indicated.

In the present study, we addressed the question of whether the loss of a single *Ibtk* allele could still have some effects on lymphoma growth. The reduced *IBT*K gene expression was confirmed in *Ibtk^+/-^ Eμ-myc* compared to *Ibtk^+/+^Eμ-myc* mice, as measured by real-time PCR (Figure 1A,B). At the macroscopic level, a significant increase in the weight and volume of lymph nodes (Figure 1C,D,E) and spleen (Figure 1F,G,H) was observed in a cohort of 12- to 16-week-old *Ibtk^+/-^Eμ-myc* mice compared to *Ibtk^+/+^Eμ-myc* mice, after tumor onset. We also observed the increased vascularization and hemorrhages of tumor lymph nodes of *Ibtk^+/-^Eμ-myc* compared to *Ibtk^+/+^Eμ-myc* mice (Figure 1E).

By flow cytometry, we performed tumor immunophenotyping of lymphoma cells using B220, IgM, and IgD as markers of B-cell subpopulations. *Ibtk^+/+^Eμ-myc* mice developed 60% pre-B lymphoma (B220^+^IgM^−^IgD^−^), 35% mature B lymphoma (B220^+^IgM^+^IgD^+^), and 5% pre-B/B lymphomas (Table 1), which was consistent with the tumor immunophenotype reported by previous studies [13,23,24]. Otherwise, *Ibtk^+/-^Eμ-myc* mice showed a significant increase of pre-B lymphoma (96%) and a decrease of mature B lymphoma (1%) (Table 1). These results indicate that the loss of a single *Ibtk* allele raises the size of Myc-driven lymphoma, with a significant impact on the tumor immunophenotype, with pre-B cell lymphoma being a more aggressive lymphoma.

**Figure 2:**
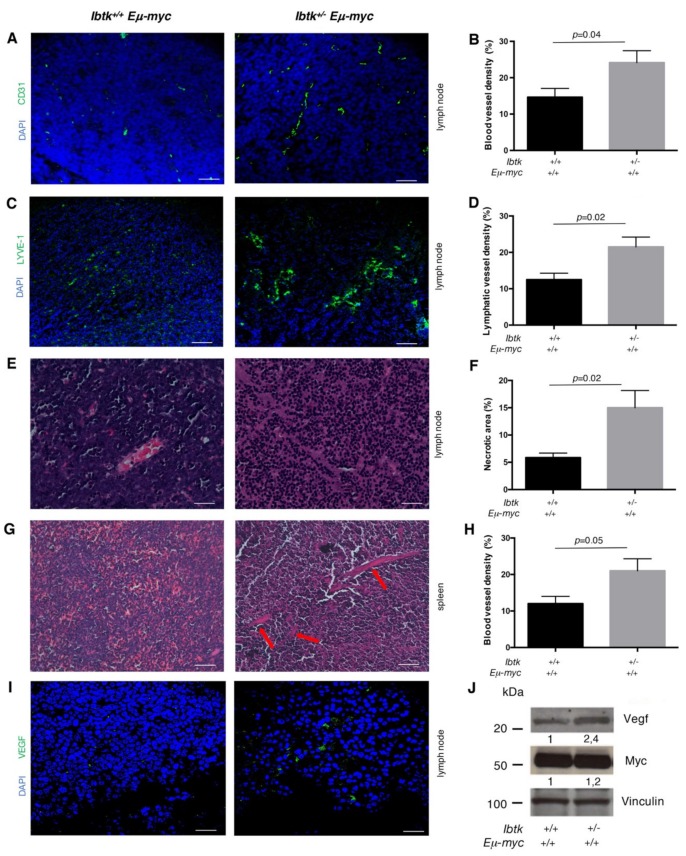
*IBTK* haploinsufficiency results in increased tumor vascularization and tissue necrosis. **A.** Sections of tumor lymph nodes of *Ibtk^+/+^Eμ-myc* and *Ibtk^+/-^ Eμ-myc* mice stained for CD31 (green) to visualize blood vessels, and analyzed by confocal microscopy. A representative image is shown. Scale bars: 150 µm. Magnification 200×. **B.** Quantitation of blood vessel density in cancerous lymph nodes of *Ibtk^+/+^ Eμ-myc* (*n* = 6) and *Ibtk^+/-^ Eμ-myc* (*n* = 6) mice. Values are the mean ± SEM. **C.** Sections of tumor lymph nodes of *Ibtk^+/+^ Eμ-myc* and *Ibtk^+/-^ Eμ-myc* mice stained for LYVE-1 (green) to visualize lymphatic vessels, and analyzed by confocal microscopy. A representative image is shown. Scale bars: 150 µm. Magnification 200×. **D.** Quantitation of the lymphatic vessel density in the tumor lymph nodes of *Ibtk^+/+^ Eμ-myc* (*n* = 6) and *Ibtk^+/-^ Eμ-myc* (*n* = 6) mice. Values are the mean ± SEM. **E.** Sections of tumor lymph nodes stained by hematoxylin/eosin. Representative images of tumor tissues are shown. Scale Bars: 75 µm. Magnification 400×. **F.** Quantitation of necrosis levels was estimated as a percentage of the total area of tumor lymph nodes of *Ibtk^+/+^ Eμ-myc* (*n* = 6) and *Ibtk^+/-^ Eμ-myc* (*n* = 6) mice. Values are the mean ± SEM. **G.** Spleen sections of sick *Ibtk^+/+^ Eμ-myc* and *Ibtk^+/-^ Eμ-myc* mice stained by hematoxylin/eosin. Red arrows indicate marked vascularization in *Ibtk^+/-^ Eμ-myc* mice. Scale bars: 150 µm. Magnification 200×. **H.** Quantitation of the vessel density in the spleen of *Ibtk^+/+^ Eμ-myc* (*n* = 6) and *Ibtk^+/-^ Eμ-myc* (*n* = 6) sick mice. Values are the mean ± SEM. **I**. Sections of tumor lymph nodes of *Ibtk^+/+^ Eμ-myc* and *Ibtk^+/-^ Eμ-myc* mice stained for VEGF (green) and analyzed by confocal microscopy. A representative image is shown. Scale Bars: 50 µm. Magnification 630 ×. **J.** Immunoblot analysis of VEGF, Myc, and vinculin expression in the protein extracts of B cells isolated from the tumor lymph nodes of *Ibtk^+/+^ Eμ-myc* and *Ibtk^+/-^ Eμ-myc* mice. Protein bands were normalized to the corresponding vinculin intensity.

Consistent with the increased vascularization (Figure 1E), the vascular density of tumor lymph nodes was higher in *Ibtk^+/-^Eμ-myc* than *Ibtk^+/+^Eμ-myc* mice, as shown by immunofluorescence staining of the blood vessel specific marker CD31 (Figure 2A,B). The number of lymphatic vessels was also increased in the tumor lymph nodes of *Ibtk^+/-^Eμ-myc* compared to *Ibtk^+/+^Eμ-myc* mice, as stained with the lymphatic vessel marker LYVE-1 (Figure 2C,D). A massive presence of necrotic areas was observed in the tumor lymph nodes of *Ibtk^+/-^Eμ-myc* compared to *Ibtk^+/+^Eμ-myc* mice (Figure 2E,F). By hematoxylin/eosin staining, we also confirmed increased vascularization in the spleen of *Ibtk^+/-^Eμ-myc* compared to *Ibtk^+/+^Eμ-myc* mice (Figure 2G,H). These results indicate that *IBTK* haploinsufficiency increases the vascularization of the tumor lymph nodes and spleen in *Eμ-myc* mice, which is associated with severe tumor necrosis. Previous studies on young preneoplastic and tumor Eμ-myc mice showed increased growth of functional blood and lymphatic vessels in hematolymphoid tissues relative to normal littermate control mice, with this effect being partly dependent on VEGF production by B lymphocytes [16]. Thus, we analyzed the VEGF expression level in lymphoma tissues by immunofluorescence. VEGF was highly expressed in the tumor lymph nodes of *Ibtk^+/-^Eμ-myc* compared to *Ibtk^+/+^Eμ-myc* mice (Figure 2I). By immunoblotting, we observed a significant VEGF increase in the protein extracts of B-cells isolated from the tumor lymph nodes of *Ibtk^+/-^Eμ-myc* compared to *Ibtk^+/+^Eμ-myc* mice (Figure 2J). Altogether, these results could indicate that the loss of a single *Ibtk* allele enhances the production of VEGF in B lymphoma cells, as a mechanism to induce vessel growth. 

### 2.2. IBTK Haploinsufficiency Increases the Production of Pro-Angiogenic Factors and Cytokines in the Tumor Microenvironment of Eμ-myc Mice

**Figure 3:**
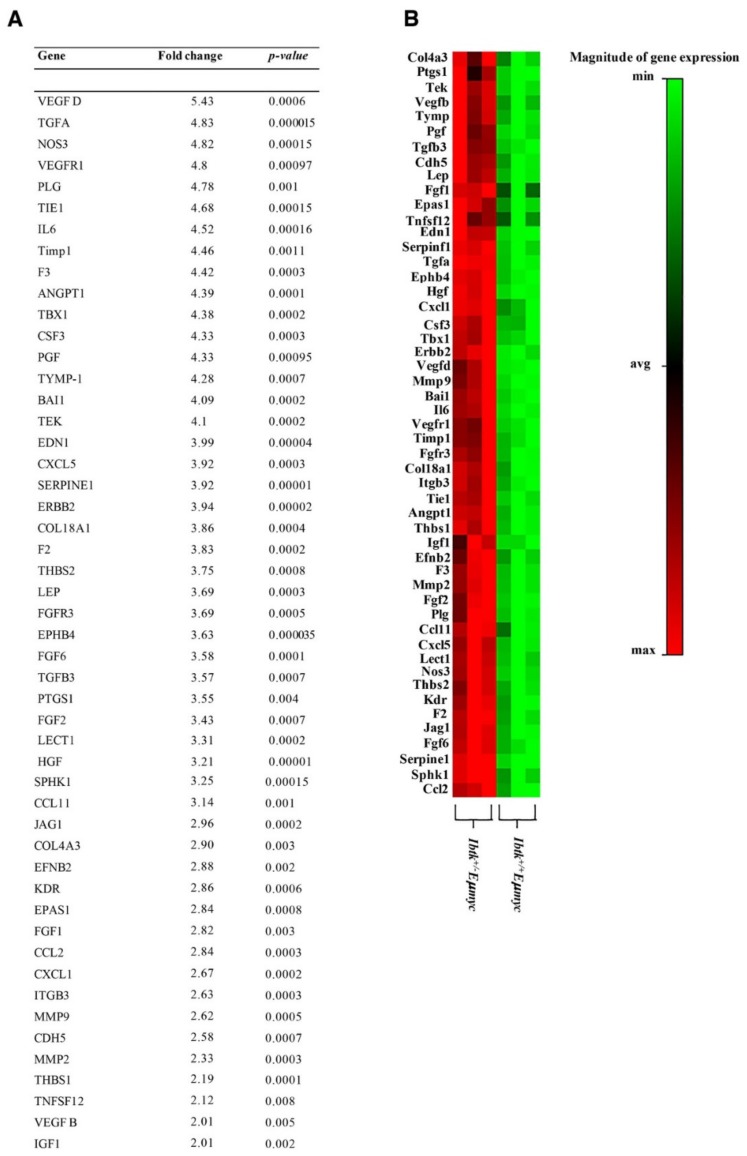
Increased expression of pro-angiogenic genes in the tumor lymph nodes of *Ibtk^+/-^ Eμ-myc* mice. **A.** Total RNA was extracted from the tumor lymph nodes of *Ibtk^+/+^ Eμ-myc* and *Ibtk^+/-^ Eμ-myc* mice, and analyzed by quantitative real-time PCR using SABiosciences Angiogenesis RT^2^ Profiler RT-PCR array. A difference in gene expression was accepted at more than a 2-fold increase. Upregulated genes are indicated as the fold change of *Ibtk^+/-^ Eμ-myc* relative to *Ibtk^+/+^ Eμ-myc* mice. Data were analyzed by two-tailed unpaired Student’ *t* test (*p* value < 0.05). **B.** Heat-map of the angiogenesis-related gene expression profile in *Ibtk^+/−^ Eμ-myc* compared to *Ibtk^+/+^ Eμ-myc* tumor B-cells.

**Figure 4:**
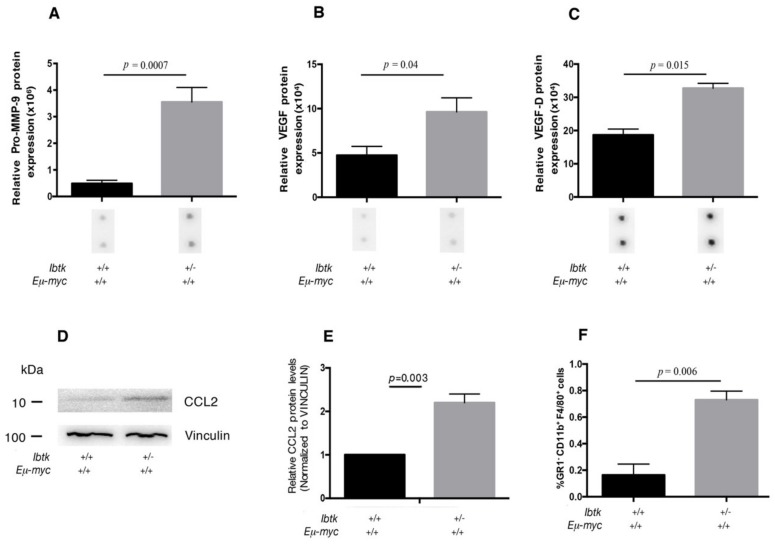
Cytokine expression and recruitment of tumor-associated macrophages in the tumor lymph nodes of *Ibtk^+/+^ Eμ-myc* and *Ibtk^+/-^ Eμ-myc* mice. **A, B, C.** Bar diagram showing the quantification of pro-MMP9, VEGF, and VEGF-D protein expression levels from *Ibtk^+/+^ Eμ-myc* and *Ibtk^+/-^ Eμ-myc* cancerous mice. Densitometry data are extracted, the background was subtracted, and the data were normalized to the positive control signals, according to the manufacturer’s protocols. Values are the mean ± SEM (*n* = 3/genotype). Representative images of individual cytokine spots are shown from identical exposures. **D.** Immunoblot analysis of CCL2 and vinculin expression in the protein extracts of tumor cells from *Ibtk^+/+^ Eμ-myc* and *Ibtk^+/-^ Eμ-myc* mice. Protein bands were normalized to the corresponding vinculin intensity. **E.** Bar diagram showing quantitation of CCL2 protein. Protein bands were measured by densitometry as arbitrary units and normalized to vinculin as the internal control. Values are the mean ± SEM (*n* = 3/genotype). **F.** Cell suspensions of tumor lymph nodes were stained with fluorescent-conjugated antibodies to reveal tumor-associated macrophages (GR1- CD11b+ F4/80+ cells) and analyzed by flow cytometry. Values are the mean ± SEM (*n* = 3/genotype).

Tumor-associated angiogenesis is a complex process, involving many proangiogenic factors, such as cytokines and chemokines. Thus, we asked the question of whether the haploidy of *Ibtk* could affect the variation of the expression of angiogenic factors on tumor cells. To this end, we analyzed the mRNA expression of 84 genes involved in the angiogenesis pathway by quantitative real-time PCR of total RNA extracted from the tumor lymph nodes of both genotypes (Table S1). The expression of 51 genes was significantly upregulated in *Ibtk^+/-^ Eμ-myc* mice, including VEGFD, VEGFR1 (as a receptor of VEGF family proteins), MMP9, and CCL2 (Figure 3A,B). Noteworthy, the highest expressed cytokine observed was VEGFD. This is a vascular endothelial growth factor previously identified as one of the predominantly lymphangiogenic factors, inducing lymphangiogenesis in transgenic mouse models [25]. This result confirms increased tumor lymphatic vascularization of *Ibtk^+/-^ Eμ-myc* compared to *Ibtk^+/+^ Eμ-myc* mice as shown by immunofluorescence with the specific lymphangiogenic marker Lyve1 [26] (Figure 2C). The enhanced expression of pro-angiogenic factors was consistent with the increased vascularization of tumor lymph nodes of *Ibtk^+/-^ Eμ-myc* compared to *Ibtk^+/+^ Eμ-myc* mice. Next, we extended our study to the tumor microenvironment. The latter includes various cell types, extracellular matrix, growth factors, proteolytic enzymes, and signaling molecules, all of which contribute to cancer development and progression. In particular, the chronic inflammation driven by chemokines and cytokines at the tumor site promotes tumor progression [27]. Thus, we sought to evaluate whether the loss of a single *Ibtk* allele could affect chemokine and cytokine expression in the tumor microenvironment of Myc-dependent lymphomas. By cytokine array, we tested the expression levels of 31 cytokines in the protein lysates of lymphomas (Table S2). We confirmed that Pro-MMP9, VEGF, and VEGFD were the cytokines more significantly increased in *Ibtk^+/-^ Eμ-myc* compared to *Ibtk^+/+^ Eμ-myc* mice. (Figure 4 A–C) (Table S2). Among the cytokines related to the tumor microenvironment, these are mainly involved in angiogenesis, promoting tumor growth [28,29]. Previous studies showed that CCL2 regulates the trafficking of monocytes, macrophages, and other inflammatory cells [30]. CCL2 also promotes the recruitment of tumor-associated macrophages (TAMs) that may secrete VEGF and the proteolytic enzyme MMP-9, which is involved in the vascularization of tumor tissues [31]. TAMs act through the promotion of angiogenesis and the facilitation of tumor metastasis [32]. Consequently, we confirmed the presence of CCL2 in lymphoma tissues by immunoblotting. We observed both increased CCL2 mRNA (Figure 3A,B) and protein (Figure 4 D) expression from tumor lymph nodes of *Ibtk^+/-^ Eμ-myc* compared to *Ibtk^+/+^ Eμ-myc* mice. We sought to assess whether the increased production of CCL2 in *Ibtk^+/-^ Eμ-myc* lymphomas could affect the presence of TAMs. By flow cytometry, a significant increase of TAMs in lymphoma of *Ibtk^+/-^ Eμ-myc* compared to *Ibtk^+/+^ Eμ-myc* mice was observed (Figure 4E). 

Taken together, these results suggest the potential involvement of IBTK in both enhanced tumor angiogenesis and TAM infiltration within the tumor.

## 3. Discussion 

In this study, we analyzed the effect of *IBTK* haploinsufficiency in B-lymphoma by taking advantage of *Eμ-myc* transgenic mice, a preclinical model of non-Hodgkin’s lymphoma. We showed that the loss of a single *Ibtk* allele increases the tumor mass and affects the tumor microenvironment of *Eμ-myc* mice. *IBTK* haploinsufficiency induces enlargement of the spleen and lymph nodes, together with an increased rate of pre-B cell lymphoma. Additionally, we observed a significant increase in pro-angiogenic and lymphangiogenic cytokines associated with TAM infiltration of lymphomas in *Ibtk^+/−^ Eμ-myc* compared to *Ibtk^+/+^ Eμ-myc* mice. 

In previous reports, both young pre-neoplastic and older tumor *Eμ-myc* mice showed increased growth of functional blood and lymphatic vessels of hemato-lymphoid tissues relative to their normal littermate control mice. Moreover, the enhanced vascularization was associated with VEGF production by B-lymphocytes, suggesting a tumor-dependent mechanism of vascularization mediated by VEGF [16]. Here, we report that the allelic deletion of *Ibtk* raises the vascularization of tumor lymph nodes and spleen, as shown by immunohistological staining. This effect was associated with an increased production of VEGF as measured by the immunofluorescence of lymphoma tissues and Western blotting of tumor B cells. Altogether, these data suggest that *IBTK* haploinsufficiency contributes to upregulating VEGF expression and, consequently, promoting vessel growth in Myc-dependent lymphoma. We also observed an increased necrosis of tumor lymph nodes of *Ibtk^+/-^ Eμ-myc* compared to *Ibtk^+/+^ Eμ-myc* mice. These findings are in agreement with previous reports, correlating necrosis of breast carcinoma with total vessel counts in highly vascularized tumor areas [33].

In addition to VEGF, the lymphoma tissues of *Ibtk^+/-^ Eμ-myc* mice hyperexpressed other pro-angiogenic factors that could induce vascularization. In particular, the expression of chemokine CCL2 was significantly elevated in the B-lymphoma cells of *Ibtk^+/-^ Eμ-myc* mice. CCL2 is a main monocyte-recruiting chemokine that acts through binding to the CCR2 receptor expressed on immune cells [34]. The enhanced expression of CCL2 was reported in many types of cancer, including multiple myeloma, melanoma, esophageal, gastric, colorectal, lung, breast, ovary, and prostate cancer [30,34]. Of note, CCL2 contributes to angiogenesis by attracting M2-like macrophages, so-called TAMs, which secrete pro-angiogenic cytokines, such as VEGF and the proteolytic enzymes, MMP-2 and MMP-9 [34,35,36,37]. According to these observations, we found an increased production of CCL2 that we could associate with the increased presence of TAMs in the lymphomas of *Ibtk^+/-^ Eμ-myc* compared to *Ibtk^+/+^ Eμ-myc* mice. Thus, the increased lymphoma vascularization of *Ibtk^+/-^ Eμ-myc* mice could be a consequence of the upregulated CCL2/TAMs/VEGF network. In this regard, the correlation between TAM infiltration and angiogenesis was previously shown in different human cancers, including breast cancer, melanoma, glioma, gastric cancer, B-cell non-Hodgkin’s lymphoma, mucoepidermoid carcinoma of salivary glands, and leiomyosarcoma, and it was associated with poor clinical outcomes [38]. Based on our observations in *Ibtk^+/-^ Eμ-myc* mice, *IBTK* could represent a novel modifier gene of the tumor microenvironment, acting through the deregulation of angiogenic and inflammatory factors. 

A deeper knowledge of the interaction between cancer cells and the microenvironment is critical for understanding tumor pathogenesis and potential new therapeutic targets. Different strategies have been developed to reduce angiogenesis to counteract tumor progression. For example, VEGF-dependent pathways have been considered a target of tumor therapy [39]. However, while some cancer types have shown an effective therapeutic response, the benefits of anti-angiogenic agents have been revealed to be transient and followed by tumor relapse [39,40,41]. With *IBTK* being required for cancer B cell survival [21,22] and lymphoma vascularization (this study), the combination of *IBTK*-targeting therapies with anti-angiogenic agents could improve the therapeutic efficacy. 

## 4. Materials and Methods

### 4.1. Mice

*Eμ-myc* transgenic mice (TgN(IghMyc)22Bri/J) were obtained from The Jackson Laboratory (Bar Harbor, Maine; USA). *Ibtk*^+/−^ mice were obtained by mating *Ibtk*^+/+^ and *Ibtk*^−/−^ mice [22]. Subsequently, *Eμ-myc* mice were crossed with *Ibtk*^+/−^ mice to generate *Ibtk^+/+^ Eμ-myc* and *Ibtk^+/−^ Eμ-myc* littermates. Mice were monitored daily for signs of morbidity and tumor development. Moribund mice and mice with obvious tumors were sacrificed, and single-cell suspensions were obtained from tumor tissues and frozen in 10% DMSO for RNA and protein analyses. 

The *Eμ-myc* transgene was detected as a 600-bp product by genomic PCR amplification, according to a previously described protocol [42]. Genotyping of *Ibtk* and *βgeo* genes was performed with primers 5′**-**GATGTAAAGCCGTGGGAGAA-3′ and 5′-ATGTGGAGAGGAGGCAGAGA-3′ (800bp product), and 5′**-**GATGTAAAGCCGTGGGAGAA-3′ and 5′-CACTCCAACCTCCGCAAACTC-3′ (500bp product), respectively [22]. Mice were sacrificed at 6–8 weeks after tumor onset, and the volume of lymph nodes was measured by the length and width. The lymph node volume was calculated by the formula: Length × Width^2^/2, and expressed as mm^3^ [43]. The volume of the spleen was calculated by the formula: Length × Width × Height, and expressed as mm^3^ [44]. The Bioethical Committee of the University Magna Graecia of Catanzaro approved the experimental protocols. Animal experiments were carried out in accordance with the protocol n.794/2016-PR approved by the Italian Ministry of Health.

### 4.2. Histological Analysis

For analysis of the intra-tumoral micro vessel density, 10-μm tissue sections were obtained by cryostat (Leica Biosystems Inc., Buffalo Grove, IL USA), incubated with monoclonal anti-CD31 antibody (BD Biosciences, San Jose, CA, USA), anti-VEGF (Santa-Cruz, TX, USA), anti-LYVE1 (BD Biosciences), and DAPI (SIGMA, St. Louis, MO, USA), and examined by confocal microscopy (Leica TC-SP2) [45]. Microvessel density within tumor sections was determined at a 200× magnification in five fields of each tumor section, as described [46]. Microvessel density was reported as the mean number of microvessels per field (56.25 μm^2^). For the histopathological analysis, lymph nodes and spleen were formalin-fixed and embedded in paraffin, and 5-μm tissue sections were stained with hematoxylin and eosin. Tissue sections were examined with a Leica Microscope (Leica Microsystems GmbH, Wetzlar, Germany) equipped with 10×, 40×, and 100× objective lenses. 

### 4.3. Western Blot Analysis and Cytokine Antibody Arrays

Cells and tissues were lysed in ice-cold modified RIPA buffer (10 mM Tris-HCl, pH 7.5, 150 mM NaCl, 1 mM EDTA, 1% Igepal), as previously described [47]. Protein samples were separated by electrophoresis on NuPAGE 4%–12% polyacrylamide gel (Thermo Fisher Scientific, Waltham, MA, USA) and transferred to a nitrocellulose membrane (BioRad, Hercules, CA, USA). Equal amounts of protein were Western blotted using the following antibodies: Myc (#5605, Cell Signaling Technology, Massachusetts, USA), VEGF (sc-507, Santa-Cruz, Texas, USA), CLL2(#2029, Cell Signaling Technology, Massachusetts, USA), and Vinculin (V9131, Sigma-Aldrich). For the cytokine array, whole tumor lysates were collected to test the cytokine levels as outlined in the mouse cytokine C-Series antibody array protocol (RayBiotech, Georgia, USA). Relative cytokine contents were quantified using Uvitec densitometry software. Briefly, after the raw numerical densitometry data were extracted, the background was subtracted and the data were normalized to the positive control signals, according to the manufacturer’s protocols. Data were analyzed using the RayBio Analysis Software Tools. 

### 4.4. Real Time PCR, Data Normalization, and Analysis

Total RNA was isolated from the tumor lymph nodes of *Ibtk^+/+^ Eμ-myc* or *Ibtk^+/-^ Eμ-myc* mice using GenElute Mammalian Total RNA Miniprep reagent (Sigma). After DNase treatment, cDNA was synthesized by an RT^2^ First Strand kit (SABiosciences. MD, USA) according to the company’s instructions. Gene expression profiling was performed using the Angiogenesis RT^2^ Profiler PCR Array (SABiosciences, MD, USA). This platform is designed to profile the expression of 84 genes of angiogenesis; for a comprehensive list of genes, see http://www.sabiosciences.com. Quantitative reverse transcriptase PCR (RT-PCR) was performed using an iScript RT-PCR System (Biorad, CA, USA) according to the manufacturer’s instructions. Relative gene expression was determined using the ΔΔCt method. Data were further analyzed using the PCR Array Data Analysis Web Portal (http://www.SABiosciences.com/pcrarraydataanalysis.php). The genes β2-microglobulin (B2M), Heat shock protein 90 alpha (cytosolic), class B member 1 (Hsp90ab1), glucuronidase-β (Gusb), glyceraldehyde-3-phosphate dehydrogenase (GAPDH), and β-actin (ACTB) were included in the PCR array as endogenous controls for normalization. Each replicate cycle threshold (CT) was normalized to the average CT of the five endogenous controls. The following formula was used to calculate the amount of transcripts in the samples relatively to the control group, both of which were normalized to the endogenous controls: ΔΔCt = ΔCt (*Ibtk^+/-^ Eμ-myc* as test sample) -ΔCt (*Ibtk^+/+^ Eμ-myc* as control sample). ΔCt is the log2 difference in Ct between the test sample gene and the control sample by subtracting the average Ct of control genes from each replicate. The fold-change of each sample relative to the control sample = 2 ^-^^ΔΔCt^. The gene expression change between *Ibtk^+/-^Eμ-myc* and *Ibtk^+/+^ Eμ-myc* tumor cells was reported as a fold increase or decrease. The adopted criteria included the following: a) Student’s *t* test with *p* value < 0.05; and b) mean difference equal to or greater than 2-folds change in expression levels. The statistical analysis was based on the web-based program for Profiler TM PCR Array Data Analysis. Data were evaluated as the mean of at least three independent experiments. 

### 4.5. Isolation of B Cells

The separation of B cells was performed by depletion of non-B cells using magnetic-activated cell sorting (MACS) B-cell isolation kit or by CD19 MicroBeads and MS columns (Miltenyi Biotech, Bergisch Gladbach, Germany) according to the manufacturer’s protocols. As a control, flow cytometry of MACS-separated cells revealed a 95% purity of B cells.

### 4.6. Flow Cytometry

Lymphoma immunophenotype was assessed as previously described [22]. The following antibodies were used for staining: CD19-APC, B220 (CD45R)-FITC, IgM biotin, IgD-PE, and Streptavidin-APC/Cy7 (BD Biosciences, USA). Data were collected by a flow cytometer (BriCyteE6, Mindray Bio- Medical Electronics Co. Ltd., Shenzhen, China) and analyzed using FlowJo Version 10.1 software. For the analysis of tumor-associated macrophages, cell suspensions of lymph nodes were obtained by grinding and filtering tissues through 0.4-μm cell strainers (BD Biosciences, USA) in PBS. Cells were then transferred to a fresh tube for centrifugation at 1000× *g* for 5 min. The cell pellet was incubated in red blood cell lysis buffer (Lonza) for 1 min at room temperature, diluted in PBS, and centrifuged for 1000× *g* for 5 min. The cell pellet was incubated with fluorescent-conjugated antibodies (dilution 1:50 in PBS) for 15 min at 4 °C in the dark, washed, and analyzed by flow cytometry. The following antibodies were used: F4/80 (6F12) from Miltenyi; CD11b (M1/70.15.5) and Gr1 (RB6–8C5) from BD Pharmingen. 

### 4.7. Statistical Analyses

Statistical analysis was performed by the two-tailed unpaired Student’s *t* test using the GraphPad Prism^®^ software package. Statistical significance was determined by *p* < 0.05 [48].

## Figures and Tables

**Table 1:** Immunophenotype of lymphomas developed by *Ibtk^+/+^ Eμ-myc* and *Ibtk^+/-^ Eμ-myc* mice.

Genotype	Pre-B cell	Pre-B/B cell	B cell
	lymphoma	lymphoma	lymphoma
*Ibtk^+/+^ Eμ-myc* (*n* = 20)	12 (60%)	1 (5%)	7 (35%)
*Ibtk^+/-^ Eμ-myc* (*n* = 24)	23 (96%)	0 (0%)	1 (4%)

Cell suspensions from lymphomas were stained with the antibodies against B220, IgM, and IgD, and analyzed by flow cytometry.

## Supplementary Materials

Supplementary Materials can be found at https://www.mdpi.com/1422-0067/21/3/885/s1.
