# Supplementary material for: IBTK Haploinsufficiency Affects the Tumor Microenvironment of Myc-Driven Lymphoma in E-myc Mice
Source: Int J Mol Sci. 2020 Jan 30;21(3):885. doi: 10.3390/ijms21030885 (PMC7038122; doi:10.3390/ijms21030885)
Supplement: Supplementary file 1 [file ijms-21-00885-s001.pdf]

Table S1. Gene expression of pro-angiogenic factors in tumor lymph nodes of *Ibtk*$^{+/+}$*Eμ-myc* and *Ibtk*$^{+/-}$*Eμ-myc* mice.

Symbol	Gene	Fold change	*p-value*
Akt1	Thymoma viral proto-oncogene 1	1,8967	0,007 061
Ang	Angiogenin, ribonuclease, RNase A family, 5	1,1159	0,929 481
Angpt1	Angiopoietin 1	4,3916	0,000 117
Angpt2	Angiopoietin 2	0,7478	0,461 625
Anpep	Alanyl (membrane) aminopeptidase	1,1015	0,258 737
Bai1	Brain-specific angiogenesis inhibitor 1	4,0927	0,000 202
Ccl11	Chemokine (C-C motif) ligand 11	3,1381	0,001 149
Ccl2	Chemokine (C-C motif) ligand 2	2,8407	0,000 298
Cdh5	Cadherin 5	2,5849	0,000 744
Col18a1	Collagen, type XVIII, alpha 1	3,8568	0,000 388
Col4a3	Collagen, type IV, alpha 3	2,9031	0,003 327
Csf3	Colony stimulating factor 3 (granulocyte)	4,3332	0,000 258
Ctgf	Connective tissue growth factor	1,0195	0,693 88
Cxcl1	Chemokine (C-X-C motif) ligand 1	2,67	0,000 21
Cxcl2	Chemokine (C-X-C motif) ligand 2	0,7507	0,067 631
Cxcl5	Chemokine (C-X-C motif) ligand 5	3,921	0,000 328
Edn1	Endothelin 1	3,9931	0,000 042
Efna1	Ephrin A1	1,6449	0,001 601
Efnb2	Ephrin B2	2,8858	0,002 042
Egf	Epidermal growth factor	1,726	0,000 51
Eng	Endoglin	0,2309	0,000 467
Epas1	Endothelial PAS domain protein 1	2,8421	0,000 764
Ephb4	Eph receptor B4	3,6334	0,000 035
Erbb2	V-erb-b2 erythroblastic leukemia viral oncogene homolog 2, neuro/glioblastoma derived oncogene homolog (avian)	3,9377	0,000 024
F2	Coagulation factor II	3,8295	0,000 239

F3	Coagulation factor III	4,4195	0,000<br>293
Fgf1	Fibroblast growth factor 1	2,8198	0,002<br>748
Fgf2	Fibroblast growth factor 2	3,4341	0,000<br>711
Fgf6	Fibroblast growth factor 6	3,5819	0,000<br>099
Fgfr3	Fibroblast growth factor receptor 3	3,6971	0,000<br>494
Fn1	Fibronectin 1	1,6387	0,000<br>983
Hgf	Hepatocyte growth factor	3,2145	0,000<br>011
Hif1a	Hypoxia inducible factor 1, alpha subunit	1,0027	0,919<br>779
Ifng	Interferon gamma	1,8132	0,003<br>184
Igf1	Insulin-like growth factor 1	2,0136	0,002<br>075
Il1b	Interleukin 1 beta	1,8431	0,005<br>453
Il6	Interleukin 6	4,5217	0,000<br>156
Itgav	Integrin alpha V	0,9934	0,954<br>852
Itgb3	Integrin beta 3	2,6289	0,000<br>337
Jag1	Jagged 1	2,9588	0,000<br>181
Kdr	Kinase insert domain protein receptor	2,8562	0,000<br>562
Lect1	Leukocyte cell derived chemotaxin 1	3,3089	0,000<br>182
Lep	Leptin	3,6998	0,000<br>295
Mapk14	Mitogen-activated protein kinase 14	1,0552	0,584<br>574
Mdk	Midkine	1,4593	0,008<br>314
Mmp14	Matrix metallopeptidase 14 (membrane-inserted)	0,4875	0,003<br>369
Mmp 9	Matrix metallopeptidase 9	2,6197	0,000<br>467
Mmp2	Matrix metallopeptidase 2	2,3353	0,000<br>265
Mmp 19	Matrix metallopeptidase 19	1,2039	0,051<br>209
Nos3	Nitric oxide synthase 3, endothelial cell	4,8217	0,000<br>148
Nrp1	Neuropilin 1	1,8741	0,000<br>112
Nrp2	Neuropilin 2	0,3616	0,000<br>624
Pdgfa	Platelet derived growth factor, alpha	3,042	0,000<br>036

Pecam1	Platelet/endothelial cell adhesion molecule 1	0,6192	0,028 404
Pgf	Placental growth factor	4,3268	0,000 951
Plau	Plasminogen activator, urokinase	1,752	0,006 632
Plg	Plasminogen	4,7774	0,001 024
Ptgs1	Prostaglandin-endoperoxide synthase 1	3,5558	0,004 215
Ptk2	PTK2 protein tyrosine kinase 2	0,5124	0,000 853
S1pr1	Sphingosine-1-phosphate receptor 1	1,0621	0,590 418
Serpine 1	Serine (or cysteine) peptidase inhibitor, clade E, member 1	3,9199	0,000 01
Serpinf1	Serine (or cysteine) peptidase inhibitor, clade F, member 1	1,8124	0,000 022
Smad5	MAD homolog 5 (Drosophila)	1,1689	0,287 074
Sphk1	Sphingosine kinase 1	3,2472	0,000 152
Tbx1	T-box 1	4,3758	0,000 164
Tek	Endothelial-specific receptor tyrosine kinase	4,0979	0,000 175
Tgfa	Transforming growth factor alpha	4,829	0,000 015
Tgfb1	Transforming growth factor, beta 1	0,9587	0,615 773
Tgfb2	Transforming growth factor, beta 2	1,4701	0,003 948
Tgfb3	Transforming growth factor, beta 3	3,5765	0,000 681
Tgfbr1	Transforming growth factor, beta receptor I	1,2546	0,015 422
Thbs1	Thrombospondin 1	2,1879	0,000 131
Thbs2	Thrombospondin 2	3,7471	0,000 77
Tie1	Tyrosine kinase with immunoglobulin-like and EGF-like domains 1	4,677	0,000 147
Timp1	Tissue inhibitor of metalloproteinase 1	4,4609	0,001 06
Timp2	Tissue inhibitor of metalloproteinase 2	1,2357	0,105 908
Tnf	Tumor necrosis factor	0,5302	0,001 272
Tnfsf12	Tumor necrosis factor (ligand) superfamily, member 12	2,1185	0,007 691
Tymp	Thymidine phosphorylase	4,2768	0,000 741
Vegfa	Vascular endothelial growth factor A	0,8873	0,181 935
Vegfb	Vascular endothelial growth factor B	2,00	0,053 742

Vegfc	Vascular endothelial growth factor C	1,2618	0,110 815
Vegf d - Figf	C-fos induced growth factor or vascular endothelial growth factor D	5,4282	0,000 596
Vegfr1 - Flt1	FMS-like tyrosine kinase 1 or Vascular endothelial growth factor receptor 1	4,7977	0,000 97

Total RNA was extracted from tumor lymph nodes of *Ibtk*$^{+/+}$*Eμ-myc* and *Ibtk*$^{+/-}$*Eμ-myc* mice, and analyzed for the expression of 84 genes by quantitative Real time PCR, using SABiosciences Angiogenesis RT^2 Profiler RT-PCR array. Genes were considered as "not detected" with Ct values ranging from 30 to 40 in *Ibtk*$^{+/+}$ *Eμ-myc* cancer cells. Significant difference in gene expression was considered for at least 2-fold change in *Ibtk*$^{+/-}$*Eμ-Myc* versus *Ibtk*$^{+/-}$*Eμ-Myc* mice. Statistical analysis was performed by two-tails unpaired Student's *t* test (p value < 0.05). Data are the mean of 3 independent experiments.

Table S2. Increased production of cytokines and chemokines in tumor lymph nodes of *Ibtk*$^{+/-}$ *Eμ-myc* mice.

**Protein**	**Fold change**	***p-value***
bFGF	0.77	0.36
Eotaxin-1	0.94	0.9
GCSF	0.78	0.71
IGF-1	0.92	0.86
IGF-2	0.92	0.85
IFN-gamma	0.84	0.5
IL-12 p40/p70	0.71	0.4
IL-12 p70	1.11	0.8
IL-17A	1.00	0.92
IL-17B	1.23	0.5
IL-17RB	0.86	0.67
IL-17E	0.99	0.98
IL-17F	0.90	0.58
IL-6	0.97	0.95
IL-6R	0.91	0.3
KC	0.98	0.95
LEPTIN	1.22	0.76
LEPTIN R	0.90	0.83
MCP-2	0.94	0.87
M-CSF	1.03	0.97
MMP-2	0.80	0.72
PRO-MMP-9	5.27	0.0007
RANTES	0.86	0.7
TIMP-2	0.94	0.8
TNF alpha	1.30	0.75
VEGF	1.86	0.04
VEGFR1	1.12	0.7
VEGFR2	1.07	0.86

VEGFR3	1.03	0.9
VEGF-D	1.75	0.015
GM-CSF	1.21	0.8

Whole cell extracts of tumor lymph nodes of *Ibtk*$^{+/+}$*Eμ-myc* and *Ibtk*$^{+/-}$*Eμ-myc* mice were analyzed by cytokines array (RayBiotech). Fold change of *Ibtk*$^{+/-}$*Eμ-myc* versus *Ibtk*$^{+/+}$*Eμ-myc* mice is reported. Statistically significant difference between *Ibtk*$^{+/+}$*Eμ-myc* and *Ibtk*$^{+/-}$*Eμ-myc* mice was evaluated by Student'*t* test (p value < 0.05). Data are the mean of at least 3 independent experiments.
